# Biomechanical assessment of grasping postures in individuals with rheumatoid arthritis when holding adaptive silverware

**DOI:** 10.3389/fresc.2026.1770139

**Published:** 2026-02-23

**Authors:** Max Jordon, David Levine, Jim Richards, Cindy Poole, Carolyn Padalino, Kristina Babbitt, Michael Brit

**Affiliations:** 1 Department of Physical Therapy, University of Tennessee at Chattanooga, Chattanooga, TN, United States; 2 School of Health, Social Work, and Sport, University of Central Lancashire, Lancashire, United Kingdom; 3 Department of Occupational Therapy, University of Tennessee at Chattanooga, Chattanooga, TN, United States; 4 University Rheumatology Associates, Chattanooga, TN, United States

**Keywords:** activities of daily living, adaptive equipment, environmental modifications, hand function, occupational therapy, rheumatoid arthritis

## Abstract

**Introduction:**

Utensils with built-up handles are often recommended to minimize the required finger joint angles for functional grasping to reduce pain and help individuals with Rheumatoid Arthritis (RA) overcome participation barriers. However, there is a paucity of data describing the impact of built-up handles on range of motion (ROM) requirements of the hand. Therefore, the purpose of this study was to evaluate how built-up handles of varying thickness affect hand ROM in individuals with RA.

**Methods:**

Thirty-five individuals with RA were instructed to grasp a standard handle spoon, a 1.00 inch, and a 1.50 inch built-up handle spoon and perform a simulated eating task. Electrogoniometers were used to measure the finger joint angles of the metacarpophalangeal (MCP), proximal interphalangeal (PIP), and distal interphalangeal (DIP) joints for digits 2–5.

**Results:**

In general, there were significant decreases in finger joint flexion as handle diameters increased. Twenty-nine of the thirty-five individuals selected the 1.50 inch handle to take home with the remainder choosing the 1.00 inch.

**Discussion:**

This study is the first to provide quantitative data to support the notion that the grasping postures required when using built-up handled spoons utilizes reduced finger joint angles for individuals with RA when compared to a standard handle spoon which could help providers when assessing patient needs and when designing hand orthoses. We recommend practitioners provide built-up handled utensils where possible, or educate people with RA on where to buy or how to make adaptive utensils, to assist the independence of individuals with RA.

**Conclusion:**

This paper supports the appropriate prescription and use of adaptive silverware by healthcare providers in the promotion of independence for individuals with RA.

## 1 Introduction

Rheumatoid arthritis (RA) is an autoimmune disease that affects multiple joints including the proximal interphalangeal (PIP) and metacarpophalangeal (MCP) joints of the hands (1). It is estimated that up to 2.7% of the population is diagnosed with rheumatoid arthritis, with 80%–90% of affected individuals reporting some degree of disability related to hand use (2–4). The inflammatory process causes the synovial membrane within the joints to thicken, causing synovial damage, and eventual bone erosion (5). The physiological destruction of the joints causes pain and fatigue with resulting limited range of motion, decreased strength, and overall decreased hand function for the performance of activities of daily living (ADLs) (6). The effects may manifest in numerous functional deficits related to ADLs such as eating, bathing, dressing, household chores, and leisure activities (7, 8).

Bazanski reported that a decrease in functional range of motion (FROM) of 50 degrees in the MCP joints was associated with a 24 percent increase in finger impairment (9). This is particularly problematic for individuals with RA when the wrist joint is affected, as wrist stability is essential for supporting the tendons involved in gripping and enabling effective use of the MCP joints (10). There are two measures of range of motion that are important to consider when working with patients with RA: active range of motion (AROM) which describes the joint measures an individual is able to actively achieve without assistance, and FROM which describes the necessary movement within joints to complete everyday tasks such as writing with a pen or using cutlery (11). Gracia-Ibáñez et al (12) reported that while there is no uniformity on the calculation or value of FROM measures, these measures are consistently lower than AROM values in the hand during grasping tasks and assessments. An implication of RA is that individuals often cannot complete functional tasks of daily living, as they often lose full AROM, impacting FROM. As people with RA lose ROM in their joints, they continue to lose strength due to disuse with between 5 and 28 degrees less movement within the joints of the hand compared to individuals without RA (12). This is important for rehabilitation practitioners such as occupational (OT) and physical therapists (PT) to keep in mind as they plan interventions, consider the use of adaptive equipment, and formulate goals for individuals with RA. This also lends support to the use of built-up handles to improve function as the disease progresses and AROM and FROM diminishes, resulting in decreased finger joint angles necessary to achieve effective grasping postures.

Bland et al. (13) assessed the impact of restrictive braces on individuals completing the Jebsen-Taylor test, which is a measure of fine and gross motor hand function that uses simulated ADLs. The study demonstrated that restricting the AROM in the joints of the hand has a greater impact on functional performance than restrictions in any other joint. Decreased AROM in the hands is common in people with RA and has functional implications, validating the need for compensatory intervention strategies and adaptive equipment for activities of daily living. OTs often suggest the use of different utensil grips for individuals with RA to reduce the required ROM necessary to improve participation and independence in eating tasks. Adaptive utensils often include increased handle diameters, and in healthy individuals, these have been shown to decrease the ROM needed when compared with standard utensils (14, 15). While current research supports that built-up handled utensils decrease the ROM necessary in the fingers and hands to grasp and release utensils when feeding in people without disease or disability (16), there is a lack of data on their efficacy in people with RA when utilizing adaptive equipment.

Assessing finger and hand posture to identify joint restrictions is challenging in clinical settings; however, this information is valuable for understanding and guiding rehabilitation activities and treatments involving hand motion, ultimately helping to evaluate the effectiveness of various therapeutic options (17, 18). Although there are anecdotal reports from clinicians on the benefits of adapted utensils on hand function, little or no data exists on hand and finger joint posture when using such devices and how these may interact with other assistive technology such as hand and wrist orthoses.

Therefore, the purpose of this study was to compare the grasping postures required for each joint in the hand when gripping a standard handle spoon and two adaptive spoons with built-up handles with diameters of 1.00 inches, and 1.50 inches during a simulated eating task. These sizes were selected based on clinical experience indicating that patients commonly prefer them among the commercially available options. The hypotheses were that a less flexed grasping posture would be required by people with RA in all finger joints when using built-up handles when compared to the standard handle, and that the 1.50 inch diameter handle would require a less flexed grasping posture than the 1.00 inch diameter handle. Portions of this text were previously published as part of a thesis.16.

## 2 Methods

### 2.1 Participants

Participants were recruited from an outpatient rheumatology clinic. Inclusion criteria were a diagnosis of RA and being 18 years or older.16 Exclusion criteria were any comorbidities that could further impair ROM of the hands and fingers such as osteoarthritis or psoriatic arthritis. All subjects read and signed a consent form prior to data collection, and the study was approved by the Institutional Review Board at the University of Tennessee at Chattanooga (IRB #17-117).

### 2.2 Recruitment

In this study, a convenience sample of individuals diagnosed with RA were recruited from a rheumatology outpatient clinic. All participants were diagnosed with RA by a board-certified rheumatologist with 35+ years of experience in diagnosing and treating individuals with RA. Although grip strength was not objectively measured, each participant was required to demonstrate sufficient strength to independently grasp a spoon with a standard handle to be included in the study.

### 2.3 Measurements

**Figure 1:**
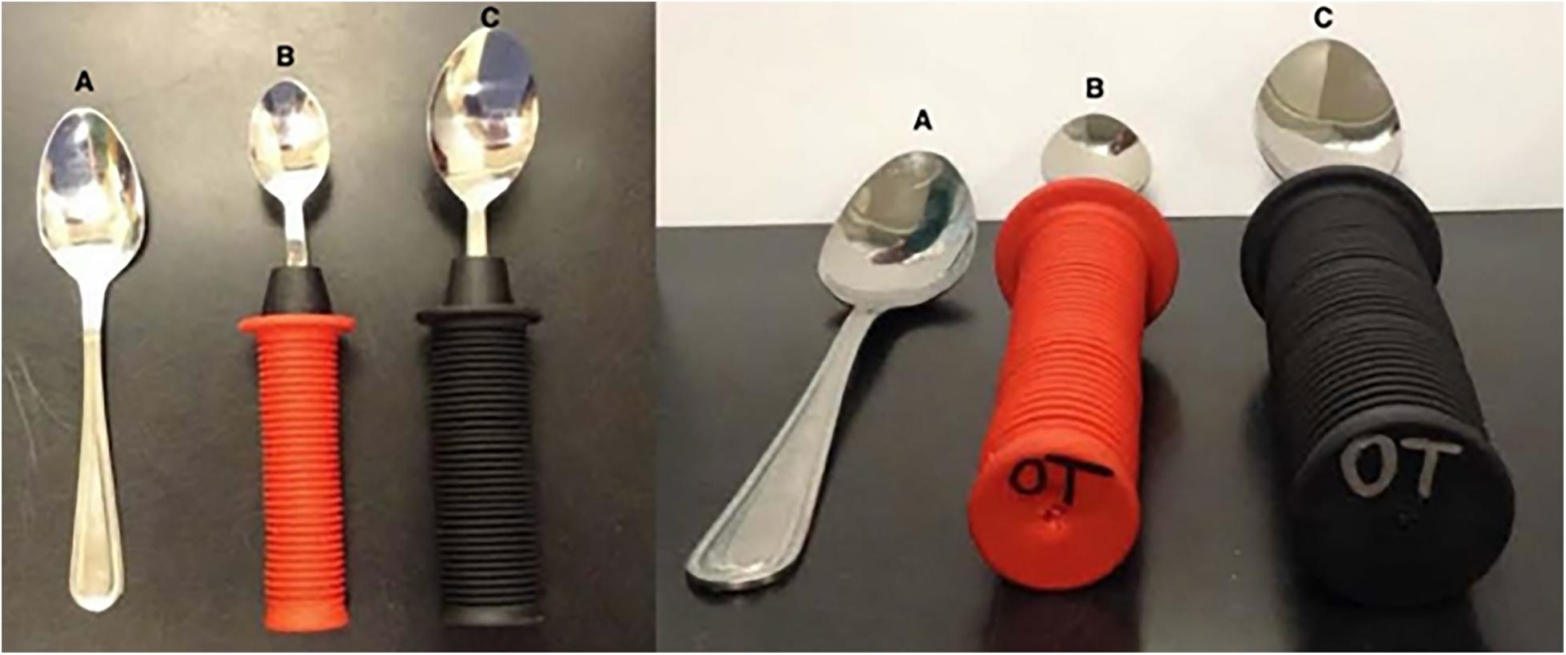
Adaptive utensils with modified handles. These images depict a standard spoon **(A)**, a spoon with a 2.54 cm (1.00 inch) diameter handle **(B)**, and a spoon with a 3.91 cm diameter handle (1.50 inch) **(C)**.

**Figure 2:**
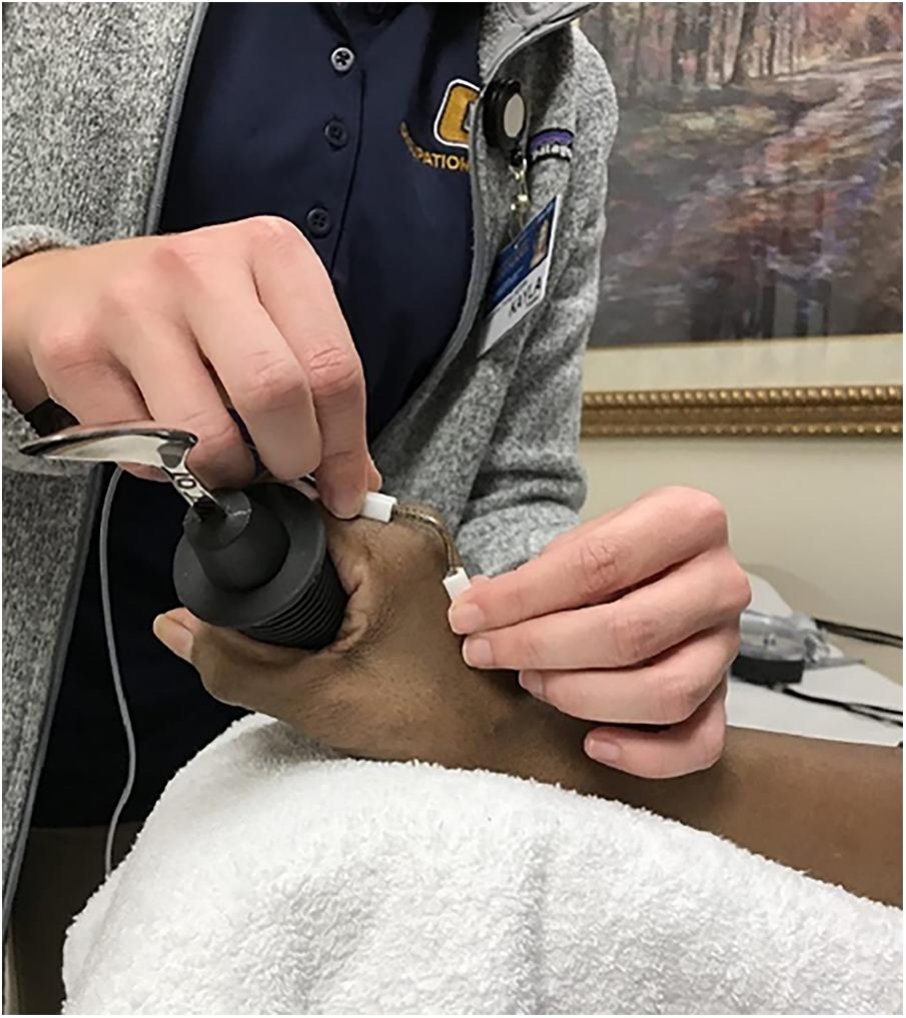
Measuring MCP flexion with the Biometrics F35 Single Axis Electrogonimeters (Biometrics Ltd, Ladysmith, VA, USA).

Measurement protocols were adapted from the study performed by McDonald et al. (14, 19) Grasping posture of all joints in digits 2 through 5 were measured bilaterally using Biometrics F35 Single Axis Electrogoniometers (Biometrics Ltd, Ladysmith, VA, USA), which have been shown to demonstrate good reliability and validity (20). Specifically for PIP flexion of the index finger, electrogoniometers have a reported ICC of 0.83 (95% CI = 0.71–0.90) and a SEM of 2.63° (20). The first digit was not included due to limitations of the measurement device, which gave abnormal readings likely caused by the positioning of the thumb on the lower surface of the spoon. Two examiners were trained in the use of the electrogoniometers and performed the measurements; however, each participant was assessed by only one examiners. The electrogoniometers were calibrated after they were attached by placing the hand on a flat surface and setting the finger joint angles to zero prior to each task, which allowed the joint angles to be calculated during the different static grasping posture tasks. Participants placed their forearm on a foam arm rest to standardize the testing position (Figure 1). The shoulder was in a comfortable amount of flexion while forearm was in a position of relative pronation with a neutral to slightly extended wrist. They were then asked to grip spoons with handles of three different diameters in a randomized order: a standard handle spoon, and built-up spoon handles with diameters of 1.00 inches, and 1.50 inches (Figure 2). Subjects were asked to hold the spoon with a cylindrical grasp, “as if you were eating cereal with milk out of a bowl.” Measurements of the metacarpophalangeal (MCP), proximal interphalangeal (PIP), and distal interphalangeal (DIP) joints were taken for digits 2–5. The cylindrical grasp was chosen as this grasp might be adopted by those with more limiting RA who would most benefit from a built-up spoon handle. Measures were repeated three times, and the average angle value for each joint was recorded. Subjects performed the task with both hands in a randomized order. Since the subjects were holding the utensil during the measurement procedure it was not possible to blind either the participant or the examiner as to which utensil was currently being grasped during the measurement. In addition, individual preference of utensil was recorded, as each participant was allowed to select one utensil to take home for their continued personal use.

### 2.4 Data analysis

Shapiro–Wilk tests were used to examine the distribution of the data, which were found to be suitable for parametric testing, and means and standard deviations were reported for all measures. The main effects of the three handle sizes (size) and the dominant and non-dominant sides (sides) were tested using a 3 (size)×2 (side) Repeated Measure ANOVA (rmANOVA) for each joint separately. For those joints where there was no significant interaction effect between size and side, the data from both sides were combined and analyzed using a one-way rmANOVA. The significant main effects of the one-way rmANOVA were further explored using Least Significant Difference (LSD) *post-hoc* pairwise comparisons. For those joints where there was a significant interaction effect between size and side, a paired t-test was performed comparing the right and left hand for each of the three sizes to determine which hand was more affected by the change in handle diameter. Effect sizes between side and size were reported using partial eta squared and the alpha level was set to 0.05.

## 3 Results

Seventy-six patients were screened with thirty-five meeting the criteria (26 female). The average age was 61.4 years (range 38–79), with an average palm width of 8.6 cm (standard deviation = 0.79). Except for the 2nd DIP joint, the 3 × 2 rmANOVAs found no significant interactions between size and side. Therefore, one-way rmANOVAs were performed for the remaining joints.

Significant main effects were seen for handle size for all measures with the greatest effects sizes typically seen in the PIP and DIP joints (Table 1). Results from the pairwise comparisons are summarized in Table 2, which shows a pattern of significant decreases in joint range of motion as handle size increased for almost all measures. The only exceptions were found in the 3rd and 4th MCP, which demonstrated no significant difference in ROM between the standard and 1in handle. Otherwise, a typical pattern of needing less ROM as the handle size increased was observed for each digit and each joint.

**Table 1:** Mean, standard deviations and main effects for repeated measures ANOVA for handle sizes (size) and dominant and non-dominant sides (side).

Joint	Dominant side (degrees) Mean (sd)			Non-dominant side (degrees) Mean (sd)			*P* value Effect size (*η*_p_^2^)	
	Standard handle	1.0-inch handle	1.5-inch handle	Standard handle	1.0-inch handle	1.5-inch handle	Side	Size
2nd digit								
MCP	75.3 (11.0)	67.4 (11.9)	62.6 (12.5)	72.3 (15.1)	71.1 (15.1)	65.6 (15.2)	0.488 (0.01)	<0.001 (0.34)*
PIP	89.9 (22.7)	75.2 (10.5)	67.6 (8.0)	90.9 (21.6)	74.9 (9.6)	68.3 (7.6)	0.827 (0.01)	<0.001 (0.59)*
DIP^Ϯ^	48.3 (11.6)	44.7 (11.5)	39.7 (10.8)	55.6 (13.0)	45.4 (10.6)	39.6 (9.8)	0.131 (0.06)	<0.001 (0.54)*
3rd digit								
MCP	81.1 (13.1)	77.5 (11.5)	73.2 (11.4)	76.8 (15.5)	77.0 (12.0)	74.8 (12.7)	0.532 (0.11)	<0.001 (0.24)*
PIP	93.9 (17.0)	76.0 (9.2)	66.5 (8.8)	97.9 (9.1)	76.1 (13.4)	66.9 (7.5)	0.281 (0.03)	<0.001 (0.81)*
DIP	56.7 (13.7)	45.6 (13.1)	41.6 (10.8)	60.6 (10.8)	48.4 (10.7)	41.3 (9.3)	0.240 (0.04)	<0.001 (0.68)*
4th digit								
MCP	77.1 (10.4)	72.1 (11.4)	69.4 (13.2)	71.1 (15.3)	72.2 (14.4)	69.5 (12.8)	0.270 (0.03)	0.003 (0.14)*
PIP	97.4 (17.1)	73.5 (9.9)	65.1 (9.7)	98.8 (9.9)	75.4 (8.6)	63.9 (8.7)	0.646 (0.01)	<0.001 (0.88)*
DIP	51.0 (14.4)	41.3 (9.9)	35.2 (9.4)	55.4 (12.7)	44.0 (11.7)	34.9 (9.6)	0.063 (0.09)	<0.001 (0.64)*
5th digit								
MCP	81.3 (12.8)	77.1 (15.2)	72.7 (14.9)	75.6 (19.7)	75.1 (16.4)	72.6 (15.0)	0.177 (0.05)	0.004 (0.14)*
PIP	86.3 (15.7)	58.0 (10.5)	46.0 (10.1)	90.8 (10.1)	57.3 (11.4)	48.0 (10.4)	0.23 (0.04)	<0.001 (0.93)*
DIP	57.3 (17.1)	42.3 (13.9)	36.6 (14.5)	60.4 (14.7)	43.3 (15.1)	34.3 (14.9)	0.774 (0.01)	<0.001 (0.76)*

sd, standard deviation; *η*_p_^2^, Partial Eta Squared.

* Significant main effect *p* < 0.05.

^Ϯ^ Significant interaction between side and handle.

**Table 2:** Pairwise comparisons of handles and sides.

Comparison	Mean difference (95% CI)	*P* value
1st digit IP		
Standard v 1 inch	−11.2 (−18.2–−4.3)*	0.002
Standard v 1.5 inch	−13.6 (−21.5–−5.7)*	0.001
1 inch v 1.5 inch	−2.4 (−6.3–1.6)	0.231
2nd PIP		
Standard v 1 inch	15.4 (10.1–20.7)*	<0.001
Standard v 1.5 inch	22.4 (17.1–27.8)*	<0.001
1 inch v 1.5 inch	7.1 (5.4–8.7)*	<0.001
3rd PIP		
Standard v 1 inch	19.9 (16.2–23.6)*	<0.001
Standard v 1.5 inch	29.2 (25.5–33)*	<0.001
1 inch v 1.5 inch	9.4 (6.6–12.1)*	<0.001
3rd DIP		
Standard v 1 inch	11.6 (8.6–14.7)*	<0.001
Standard v 1.5 inch	17.2 (13.9–20.6)*	<0.001
1 inch v 1.5 inch	5.6 (3.5–7.6)*	<0.001
4th PIP		
Standard v 1 inch	23.6 (21.1–26.2)*	<0.001
Standard v 1.5 inch	33.6 (29.8–37.5)*	<0.001
1 inch v 1.5 inch	10.0 (7.7–12.2)*	<0.001
4th DIP		
Standard v 1 inch	10.6 (7.2–13.9)*	<0.001
Standard v 1.5 inch	18.1 (14.3–21.9)*	<0.001
1 inch v 1.5 inch	7.6 (5.5–9.6)*	<0.001
5th MCP		
Standard v 1 inch	2.3 (−1.5–6.2)	0.229
Standard v 1.5 inch	5.8 (1.8–9.8)*	0.005
1 inch v 1.5 inch	3.5 (1.3–5.7)*	0.002
5th PIP		
Standard v 1 inch	30.9 (27.8–34)*	<0.001
Standard v 1.5 inch	41.5 (38.5–44.6)*	<0.001
1 inch v 1.5 inch	10.6 (8.9–12.4)*	<0.001
5th DIP		
Standard v 1 inch	16.0 (12.8–19.3)*	<0.001
Standard v 1.5 inch	23.4 (19.6–27.1)*	<0.001
1 inch v 1.5 inch	7.3 (5.2–9.4)*	<0.001
Dominant vs. non-dominant		
1st digit MCP	4.5 (0.8–8.1)*	0.017

IP, Interphalangeal; PIP, Proximal Interphalangeal; DIP, Distal Interphalangeal.

* Significant difference *p* < 0.05.

As indicated above, there was a statistically significant interaction between size and side on ROM at the 2nd DIP [*F*(2,68) = 6.188, *p* = 0.003, partial *η*2 = .154]. Paired t-tests revealed no difference in the 1in and 1.5in handle size between the hands (*p* = .967 and.498 respectively), but a significant difference between the sides for the standard size handle (*p* = 0.019). For the standard size handle, the left side had on average 5.457 degrees more flexion than the right (55.086 vs. 49.629). Pairwise comparisons for the 1in and 1.5in handle sizes revealed no differences between the sides (Table 3). Pairwise comparisons for the main effect of size demonstrated the same pattern as observed in the other joints: less ROM was needed as handle size increased (52.36, 44.73, 39.86 degrees of flexion for the standard, 1in, and 1.5in handles respectively).

**Table 3:** *Post Hoc* tests for interaction effects for handle size (handle) for the dominant and non-dominant sides.

Comparison	Dominant		Non-dominant	
	Mean difference (95% CI)	*P* value	Mean difference (95% CI)	*P* value
2nd digit MCP				
Standard v 1 inch	8.0 (4–11.9)*	<0.001	1.1 (−4.3–6.6)	0.670
Standard v 1.5 inch	12.7 (8.5–16.9)*	<0.001	6.7 (1.8–11.5)*	0.008
1 inch v 1.5 inch	4.7 (1.2–8.2)*	0.009	5.5 (3.2–7.8)*	<0.001
2nd DIP				
Standard v 1 inch	3.7 (−0.6–7.9)	0.087	10.2 (6.7–13.7)*	<0.001
Standard v 1.5 inch	8.6 (5.4–11.8)*	<0.001	16.0 (12.4–19.6)*	<0.001
1 inch v 1.5 inch	4.9 (2–7.9)*	0.002	5.8 (3.7–8)*	<0.001
3rd MCP				
Standard v 1 inch	3.6 (1–6.3)*	0.009	−0.2 (−4.3–3.9)	0.928
Standard v 1.5 inch	7.9 (4.7–11.2)*	<0.001	2.1 (−2.3–6.4)	0.345
1 inch v 1.5 inch	4.3 (2.3–6.3)*	<0.001	2.2 (0.6–3.8)*	0.007
4th MCP				
Standard v 1 inch	5.1 (1.8–8.3)*	0.003	−1.1 (−5.8–3.7)	0.654
Standard v 1.5 inch	7.7 (3.6–11.7)*	<0.001	1.6 (−3.2–6.4)	0.510
1 inch v 1.5 inch	2.6 (−0.7–5.9)	0.119	2.6 (0.6–4.7)*	0.013

* Significant difference *p* < 0.05. Values in parentheses.

When considering selection of utensil to take home for continued personal use, 29 out of the 35 individuals chose the 1.50 inch handle with the remainder choosing the 1.00 inch. This selection may have been related to palm width; however, an additional unpaired t test showed no differences in palm width when considered in relation to the selected utensils (*p* = 0.524).

## 4 Discussion

The aim of this study was to compare the finger joint angle required for each joint in the hand when gripping a standard handle spoon and two adaptive spoons with built-up handles with diameters of 1.00 and 1.50 inches during a simulated eating task in individuals with RA. It was hypothesized that, as spoon handle diameter increased, the finger joint angle required to grip the spoons would decrease. The data collected for this study supports this hypothesis, with this pattern being observed at almost every joint.

Occupational and physical therapists provide interventions which aim to reduce barriers to participation in daily tasks in a variety of ways, including use of adaptive equipment (21). Adaptive equipment, such as utensils with built-up handles, are often recommended by practitioners for people with RA. OTs may reason that by providing built-up grips on utensils, the required finger joint angles for using the utensil will reduce which should allow a person who has limited AROM, such as with RA, to better use the utensil to participate in eating tasks. However, research related to the efficacy of adaptive equipment interventions in people with RA seems lacking (16). The findings of this study provide support for the idea that adaptive and built-up utensils could be used by individuals with functional grasping posture deficits and reduced ROM in their hands and fingers to overcome these impairments.

This study provides quantitative data to support the notion that lower joint flexion angles are needed when grasping a built-up handled spoon. These results are of clinical relevance to health professionals when treating individuals with RA who have reduced FROM. The PIP joint on digits 2–5 are considered to be the most important joints for grasp (22). As demonstrated in our results, the PIP joints required the most FROM with the standard handle spoon in comparison to the built-up handle spoons. Moreover, there was a greater average difference in the finger postures required in the PIP joints when using the standard handle spoon compared to the 1.50 inch spoon. However, no differences in the finger postures required were seen between the dominant and non-dominant sides with the exception of the 2nd DIP. This contrasts with the findings of Adams et al. (23), who reported greater AROM in the dominant hand for some—but not all—digits, potentially highlighting an important distinction between FROM and grasping posture.

Although this study's inclusion criteria relative to diagnosis was limited to people with RA, the findings may be generalizable to individuals impacted by other conditions that limit AROM in the joints of the hands and fingers, such as osteoarthritis and psoriatic arthritis. Though it was not formally included in the collection of data, several participants reported increased comfort in grasping a built-up handle spoon. When considering the individual preferences of utensil for continued personal use, 29 out of the 35 individuals chose the 1.50 inch handle with the remainder choosing the 1.00 inch. One factor that could be relevant to selection is palm width, however no differences in palm width were seen when considered in relation to the selected utensils, so this appears to be due to personal preference rather than ergonomic factors. Additionally, while the average palm width for females has been reported to be slightly smaller than the average palm width for males, this did not seem to impact the results of this study.

Currently, there is limited research on the use of adaptive utensils for individuals with functional impairments, despite their potential to offer significant benefits. Future studies assessing the impact of built-up handled items on complaints of pain, fatigue and overall functional independence would further bolster support for provision of these adaptive devices to patients who present with decreased AROM due to RA. While this research focused on individuals with RA, several other diagnoses were encountered, including Dupuytren's Contracture and Psoriatic Arthritis. We recommend further investigation into the benefits of adaptive utensils as they apply to such conditions. Similarly, it can be postulated that the use of built-up handles on other common objects may also require less range of motion and result in increased comfort and adherence with less pain when grasping. Regarding future research endeavors, there is a need for evidence concerning the grip coefficients and the force dynamic that is required when using adaptive silverware. Additionally, a study comparing the functional grasp, gross grasp and associated pain in individuals with RA when using other objects for activities of daily living is highly recommended.

Limitations of this study include only measuring the ROM at the DIP and PIP while grasping a spoon and not measuring changes which might occur during the entire ROM during the whole task. This limitation means that the findings cannot be generalized to other eating utensils, such as forks or knives, nor can they be applied to ADLs like cutting. Furthermore, it takes specialized equipment to measure the ROM making replication of these findings only accessible to those with a biomechanics lab. Since this study used a sample of convenience of individuals with RA, only the broad diagnostic criteria of RA were used for recruitment purposes. This limits the interpretability of the findings as RA classifications and Larsen stages were not recorded. Future studies could include severity of RA as a factor in the analysis of digit ROM.

Additional limitations included why subjects chose a particular handle for their personal use was not examined but could have been related to comfort, a decrease in pain, the presence of the anti-slip texture, or other reasons related to disease severity or projection of future needs by the individual. As indicated, the cylindrical grasp was chosen as it might reflect that preferred grasping posture of an individual with advanced RA who has limited ROM (24, 25). However, this rational was based on clinical experience and not established literature. Future studies should collect user feedback on why participants choose the utensils that they did. Additionally, the results of this study may not be generalizable to other diagnosis affecting the hand such as osteoarthritis, as different conditions affect different joints. Further work should consider factors including palm and finger length which may have shown differences in the choice of selected utensil handle size. Additionally, as the purpose of this study was to quantify the changes in ROM, measures of function such as handgrip strength, the Disabilities of the Arm, Shoulder and Hand questionnaire, or pain, were not obtained. Inclusion of these measures in future studies could provide a more complete understanding of the functional requirements associated with different handle sizes.

## 5 Conclusions

This study offers quantitative evidence that different grasping postures are used when holding a built-up handled spoon, supporting their use for individuals with rheumatoid arthritis (RA) who experience pain using standard-handle utensils. While it highlights the potential benefits of adaptive utensils, further research is needed to explore the advantages of various design options. The findings provide insight into the grasping postures required for individuals with RA to use built-up handled spoons effectively, which may assist clinicians in assessing patient needs and in designing hand orthoses. It is recommended that practitioners offer built-up handled utensils when possible or educate individuals with RA on how to obtain or create their own adaptive utensils.

## Data Availability

The raw data supporting the conclusions of this article will be made available by the authors, without undue reservation.
